# Towards Trustworthy Energy Disaggregation: A Review of Challenges, Methods, and Perspectives for Non-Intrusive Load Monitoring

**DOI:** 10.3390/s22155872

**Published:** 2022-08-05

**Authors:** Maria Kaselimi, Eftychios Protopapadakis, Athanasios Voulodimos, Nikolaos Doulamis, Anastasios Doulamis

**Affiliations:** 1 School of Rural and Surveying Engineering, National Technical University of Athens, 15773 Athens, Greece; 2 School of Electrical and Computer Engineering, National Technical University of Athens, 15773 Athens, Greece

**Keywords:** nonintrusive load monitoring, energy disaggregation, machine learning, signal processing, review

## Abstract

Non-intrusive load monitoring (NILM) is the task of disaggregating the total power consumption into its individual sub-components. Over the years, signal processing and machine learning algorithms have been combined to achieve this. Many publications and extensive research works are performed on energy disaggregation or NILM for the state-of-the-art methods to reach the desired performance. The initial interest of the scientific community to formulate and describe mathematically the NILM problem using machine learning tools has now shifted into a more practical NILM. Currently, we are in the mature NILM period where there is an attempt for NILM to be applied in real-life application scenarios. Thus, the complexity of the algorithms, transferability, reliability, practicality, and, in general, trustworthiness are the main issues of interest. This review narrows the gap between the early immature NILM era and the mature one. In particular, the paper provides a comprehensive literature review of the NILM methods for residential appliances only. The paper analyzes, summarizes, and presents the outcomes of a large number of recently published scholarly articles. Furthermore, the paper discusses the highlights of these methods and introduces the research dilemmas that should be taken into consideration by researchers to apply NILM methods. Finally, we show the need for transferring the traditional disaggregation models into a practical and trustworthy framework.

## 1. Introduction

Environmental policies, responses, or solutions to climate change at a global scale is a prerequisite to raise awareness of individuals or social groups on protecting our world and retaining its sustainability [1]. There are various ways that householders could contribute towards sustainable living. One of them is by reducing their energy consumption. To this end, such a reduction requires a change of humans’ energy-related behavior in their households. To shape this behavioral change, consumers need to become aware of the energy they consume. However, end-consumers often lack knowledge about potential energy savings, existing policy measures, and relevant technologies. Most household consumers are only aware of general information related to their consumption through monthly electricity bills. Nonetheless, the effectiveness of feedback on energy consumption is crucial and is usually translated into good practices and tailored advice for energy savings.

Non-intrusive load monitoring (NILM) uses the aggregate power signal of a household as the input to estimate the extent to which each appliance contributes to the aggregate energy consumption signal [2]. Using NILM techniques, one can provide itemized energy bills and personalized energy savings recommendations. Thus, NILM is an efficient and cost-effective framework for energy consumption awareness. Power disaggregation is applied to enhance awareness of the energy consumption behavior of consumers in the household and, therefore, guide them towards a prudent and rational utilization of energy resources [3].

**Figure 1:**
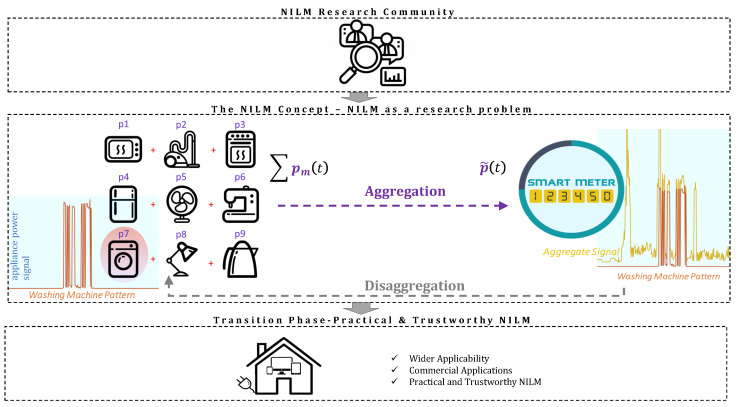
The energy disaggregation concept. Until recently, the most crucial issue was to create NILM algorithms with good performance. NILM belongs to the category of inverse problems, and formulating this mathematical problem and adapting it using machine learning models were quite challenging (early immature NILM era). Currently, given that the state-of-the-art algorithms achieve good performance, we come across the transition phase where the research interest is concentrated on practical and trustworthy NILM algorithms.

A significant number of publications and extensive research works have been carried out on energy disaggregation or NILM for the state-of-the-art methods to reach the desired performance (see Section 4). The initial interest of the scientific community to formulate and describe mathematically the NILM problem using machine learning tools—which is the main topic of interest in the review papers that are currently available—has now shifted into a more practical approach towards NILM. Currently, we are in the mature NILM period where there is an attempt for NILM to be applied in real-life application scenarios [3,4,5,6,7,8,9,10,11,12,13,14,15]. Thus, the complexity of the algorithms, transferability, reliability, practicality, and, in general, trustworthiness are the main issues of interest. This review narrows the gap between the early immature NILM era and the mature one (see Figure 1). In particular, the scope of this work is summarized as follows:

- Provides a short literature review on the existing NILM methods for residential appliances and highlights the trustworthiness aspects of the current state-of-the-art methods.
- Collects the research dilemmas that have appeared in the literature for solving the NILM problem and discusses the advantages and disadvantages of the different approaches.
- Highlights the existing challenges in NILM and discusses the barriers and limitations towards a reliable, practical, and trustworthy NILM framework.
- Discusses the future perspectives on NILM models under a trustworthy framework.

The remaining survey is structured as follows: In Section 2, we define the topic of this literature review and describe the NILM problem in residential settings, identifying the relevant gaps and challenges in the current knowledge. Section 4 synthesizes the information in the literature about NILM into a summary, organized from a chronological point of view. The brief literature review identifies the important works in the energy disaggregation area, starting from the early NILM era, until currently, when NILM researchers approach advanced NILM issues and challenges using state-of-the-art signal processing and machine learning algorithms. The remaining sections are organized to follow the general pipeline of NILM in the literature, that is: (a) signal pre-processing techniques and feature extraction and selection, (b) the machine learning part of the algorithm, and (c) load disaggregation and evaluation of the results. In particular, Section 5 is an in-depth analysis of the common signal pre-processing and feature extraction techniques available in the literature for NILM. Section 6 identifies the opposing views in machine learning techniques applied for NILM and identifies the trends emerging from the analysis and authors understanding of NILM. Section 7 constitutes an important dimension in this literature review. This section is a discussion about the trustworthiness of the NILM algorithms. Trustworthy AI has attracted immense attention recently, allowing humans to realize the full potential of AI, so that humans can fully trust and live in harmony with AI technologies. In this literature review, we discuss the key papers towards trusted NILM solutions, and we identify the future perspectives in NILM in order to build upon trustworthiness. Section 8 is a summary of the existing datasets, evaluation metrics, and open NILM tools. Furthermore, in this section, we present a critical point of view as regards the performance evaluation for the different types of residential appliances. In Section 9, possible NILM applications including energy efficiency, occupancy detection, home energy management systems and ambient assisted living, detection of transients, and applications in demand response systems for use on the smart grid are discussed. Finally, Section 10 is a discussion and a final conclusion about NILM techniques, towards efficient and trustworthy NILM algorithms during the whole NILM implementation pipeline.

## 2. Background on NILM

### 2.1. NILM Problem Formulation

Disaggregation of households’ power consumption allows grid operators to improve their predictions in energy demand and is an important part of providing a stable supply of power to all customers on a power grid [16]. The consumption profile of appliances is identified through disaggregation, and then, the obtained appliance-level load profiles along with meteorological information are employed to predict the future usage, as in [16,17].

Thus, energy disaggregation is of great importance for energy conservation and planning. Given the power consumption per appliance, the forward problem is to predict the total power consumption in a household (see Figure 1). Energy disaggregation is described as an inverse ill-posed problem, which aims to estimate unknown individualized components from aggregate measurements. We assumed the aggregate signal p(t) at a discrete time index *t* to be equal to the summation of the individual appliances’ power consumption $p_{m}\left(t\right)$ plus an additive noise ϵ(t). Thus:

(1) $$p\left(t\right)=\sum_{m=1}^{M}p_{m}\left(t\right)+\epsilon \left(t\right)$$

In Equation (1), variable *m* refers to the *m*-th out of *M* available appliances. Under a NILM framework, the individual appliance power consumption $p_{m}\left(t\right)$ is not a priori available, assuming the absence of installed smart plugs. Instead, only p(t) is given. The inverse ill-posed problem, called NILM (see Figure 1), is to calculate the best estimates ${\hat{p}}_{m}\left(t\right)$ of the actual values of the appliance power consumption $p_{m}\left(t\right)$, given the aggregate power value p(t).

### 2.2. Challenges to NILM

Various approaches have been proposed to solve the NILM problem, as presented in Section 4. Some of the most successful ones exploit deep learning neural network structures for modeling an energy disaggregation problem (e.g., [18]). Nevertheless, nonintrusive load monitoring is a challenging task. We hereby provide an indicative list of the NILM challenges based on our current understanding of the field. Some of them are well-studied, whereas others are immature and there is ongoing research in these topics:

“Challenge 1: To create reliable algorithms with good generalization ability”:

Most state-of-the-art techniques have not been applied successfully in unseen houses (transferability), across different households and datasets [19]. Therefore, it is difficult to create reliable algorithms with a good generalization ability. Large-scale trials comprise a necessary step towards this direction. Another aspect of NILM problem is to create robust models and deal with noisy datasets and appliances with abnormal behavior. Noisy aggregate energy consumption measurements significantly deteriorate the performance of NILM methods. In addition, a common problem in NILM is that the targeted appliances have unsteady signatures or present abnormal behavior. On top of these barriers, inadequate datasets deteriorate the overall models’ performance [20].

“Challenge 2: To develop hybrid NILM models incorporating user’s feedback and techniques that support continuous learning”:

Consumers’ habits and seasonality significantly affect the energy usage patterns and introduce an additional challenge in load monitoring. Various factors, including environmental, socioeconomic factors, etc., affect the operation of various domestic appliances. Users’ feedback in NILM algorithms is crucial in order to improve the models’ accuracy. Modern NILM methods should be dynamically updated and improved based on user’s recommendations.

“Challenge 3: To provide explainable NILM models with reasoning behind the model estimations”:

Even though the recently proposed models in the literature provide competitive accuracy, the inner workings of these models are less clear. Understanding and trusting the outputs of the networks help in improving the designs, highlight the relevant features and aspects of the data used for making the decision, provide a better insight into the model’s accuracy, as well as inherently provide a level of trust in the value of the provided consumption feedback to the NILM end-user.

“Challenge 4: To achieve fairness in NILM”:

The various socioeconomic, environmental, etc., factors that affect power load consumption lead to multiple distinct data distributions (e.g., geographic groups or social categories) that should be expressively modeled and represented under a NILM framework. Thus, the NILM AI framework learns to predict outcomes that are accurate with respect to the ground truth data of the target appliance used for validation, but also fair with respect to a set of pre-defined fairness metrics, leveraging sufficient and diverse training data. Except for fair data and models, fair performance evaluation that enables proper benchmarks is another important aspect for practical NILM.

“Challenge 5: To provide privacy-preserving outcomes using secure NILM models”:

To achieve the real-world applicability of NILM, we should previously address privacy concerns in NILM applications in order to provide personalized NILM services. The emerging NILM deep learning models require massive amounts of real-life data to improve their performance. Thus, data security and user privacy have become important issues.

## 3. Paper Selection Methodology

**Figure 2:**
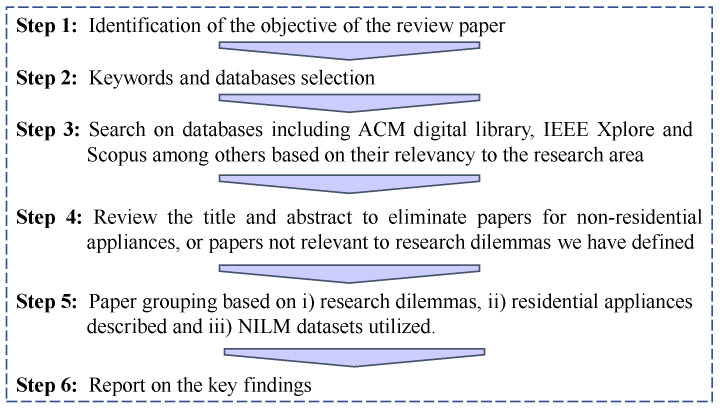
Paper selection methodology.

The process that we followed to write this review paper is summarized in Figure 2. During the design phase of this review, we conducted a comprehensive search to identify all relevant studies. We narrowed down the initial list of papers based on some criteria. The selected papers included analysis and NILM models for residential appliances, so reviews with commercial appliances were excluded from the study. Then, we grouped these studies based on the research dilemmas (appearing in Section 6.1). Furthermore, we present comparative results and a short statistical analysis of the performance achieved in some of the most recent state-of-the-art models that achieve the best performance in the literature.

## 4. A Brief NILM Literature Review

Hart [2] first introduced NILM as a method capable of estimating the energy used by individual appliances, given only the total energy consumption. At first, NILM is modeled as a linear combination problem, where each time, the algorithm estimates the percentage of the total power consumption that an active appliance consumes. The ability of collecting massive amounts of data related to household power consumption, along with the evolution of deep learning methods, made the NILM formulation as a nonlinear problem possible. Thus, we observed the pairs of data ($p_{m}\left(t\right),p\left(t\right)$), where $p_{m}\left(t\right)$ and p(t) denote, respectively, the power reading of an appliance and the mains at time *t*. Given that there are plenty of observations, it is possible to train learning models to represent the relationship between $p_{m}$ and *p* [20]. Since then, a number of studies have extended the previously simple linear model into a nonlinear one, applying various deep learning schemes.

### 4.1. The Early NILM Era (1995–2014)

Hart was the first to propose a method for disaggregating electrical loads through clustering of similar events based on appliances’ characteristics [2]. This approach employed combinatorial optimization (CO), which, at the time, was the standard technique for disaggregation problems. This first approach had a major shortcoming: combinatorial optimization performed the power disaggregation on each instant independently of the others, without considering the load evolution through time. Most common approaches to solve the NILM problem are based on unsupervised event detection in the aggregate signal, whereas supervised classifiers are used to assign known appliances to detected events in order to estimate the power trace of individual appliances. Different classification tools have been widely used, including support vector machines (SVMs) [21], neural networks, decision trees (DTs) [22], and hybrid classification methods [23,24]. Contrary to the aforementioned classic methods, other methods such as dynamic time warping (DTW) are used for comparing and grouping windows from daily profiles and identifying unique load signatures [25]. The main object of controversy in these approaches refers to the difficulty of classifying multi-state appliances [24,26]. Multi-state appliances require a long-range pattern to be trained for their detection [26]. Graph signal processing (GSP) [23] is a concept that effectively captures spatio-temporal correlation among data samples by embedding the structure of signals into a graph. Zhao et al. [27] proposed a low-resolution, event-based, unsupervised GSP approach. Recently, a modified cross-entropy method for event classification has been suggested [28], which is based on CO and formulates NILM as a cross-entropy problem.

Hidden Markov models (HMMs) and various extensions of them are advocated in order to explore the possible combinations among the different appliances’ state sequences [14,29,30,31]. In this light, HMMs are state-based, so the studied appliances should have discrete states in their signatures [32]. As the number of appliances increases, the number of combinations of state sequences is increased exponentially, increasing, respectively, the problem’s complexity [32]. In addition to this, time complexity is also increased, leading to the reduction of the model’s classification performance [26]. Makonin et al. [33] proposed a super-state HMM and a sparse Viterbi algorithm in order to reduce the complexity. Another limitation of HMM-based approaches is that they tend to fail in the presence of unknown appliances [32]. Rahimpour et al. [34] proposed a matrix factorization technique for linear decomposition of the aggregated signal using as the bases of this learned model the appliances’ signatures, resulting in an efficient estimation of the energy consumption per appliance.

### 4.2. Deep-Learning-Based NILM (2015–2019)

NILM algorithms have received renewed attention, mostly thanks to the increased number of datasets stemming from smart electric meters installed in domestic residences [35,36], and thanks to the increased number of these datasets, the proposed solutions to NILM shifted to a supervised learning process. With the rise of deep learning, a new family of methods has been introduced that exploit deep neural network structures to solve the ill-posed NILM problem. Deep learning techniques have been applied mostly to low-frequency NILM approaches since 2015 [9].

A common approach is to treat the aggregated signal as a corrupted by noise signal of an appliance. Under this view, denoising autoencoders (DAEs) are excellent techniques used to reconstruct a signal from its noisy version. This architecture was initially proposed by Kelly and Knottenbelt [9], while others expanded the idea, proposing alternative DAE architectures, such as [14].

Exploiting the temporal character and dependencies of the power signal, another family of deep learning models, recurrent neural networks (RNNs), has proven efficient under the NILM framework. Here, NILM is treated as a supervised learning problem with times series. RNNs and their variants, such as long short-term memory networks (LSTM) and gated recurrent units (GRUs), have been primarily used, as they are very popular and effective with 1D time series data. Relevant studies have been carried out in the past [9,19,26]. In a previous work of ours, we also proposed a Bayesian optimized bidirectional LSTM model for NILM [18], whereas in [37], a context-aware LSTM model adaptable to external environmental conditions was presented.

Although convolutional neural networks (CNNs) are traditionally developed for two-dimensional imagery data [38], one-dimensional CNN can be used to model the temporal character of sequential time series data. Few researchers [39] have tried to enrich CNN structures providing a recurrent character, such as CNN-LSTM and recurrent convolutional networks. In [40], a causal 1D convolutional neural network for NILM was proposed. Others introduced the concept of data sequences [9] to feed the classic structure with historical past values of power load. Others [41] propose a sequence-to-point CNN architecture, underscoring the importance of sliding windows to handle long-term time series. Alternatively, sequence-to-sequence architectures have also been proposed [42].

### 4.3. Current Advancements in NILM (2020–Present)

Recently, there have been various advanced machine learning methods applied for NILM. These methods do not only provide competitive accuracy against the traditional NILM methods, but also propose possible solutions to solve the remaining challenges in NILM and are an attempt towards a trustworthy NILM in terms of accuracy, robustness, reliability, explainability, and fairness. Some of the works worthy of mention are presented here.

Generative adversarial networks (GANs) recently have been applied for NILM. An early attempt for solving NILM using a GAN-based framework was adopted in [43]. Then, Kaselimi et al. [44] proposed a generative adversarial network for sequence-to-sequence learning, whereas Pan et al. [45] achieved sequence-to-sub-sequence learning with conditional GANs. Chen et al. [46] proposed a context-aware convolutional network for NILM that has been trained adversarially. Most of these studies exploit the robustness to noise that the adversarial training process achieves.

Transformer models were explored as an alternative architecture for neural machine translation tasks within the past two years [47]. Recently, a transformer-based architecture that utilizes self-attention for energy disaggregation was adopted by [48], to handle power signal sequential data.

Given that, most of the existing deep learning models for NILM use a single-task learning approach in which a neural network is trained exclusively for each appliance. In contrast to a single-task learning approach, the work of [49] proposes UNet-NILM for multi-task appliances’ state detection and power estimation, applying a multi-label learning strategy and multi-target quantile regression. The UNet-NILM is a one-dimensional CNN based on the U-Net architecture initially proposed for image segmentation.

Explainable AI (XAI) attempts to promote a more transparent and trustworthy AI through the creation of methods that make the function and predictions of machine learning systems comprehensible to humans, without sacrificing performance levels [50]. Explainable NILM networks proposed by [51] try to understand the inner workings of the machine learning models used for NILM.

## 5. Signal Analysis and Feature Extraction

It is experimentally proven that applying data re-sampling, data cleaning methods, and dataset balancing significantly improves energy disaggregation in terms of accuracy and generalization abilities [52]. However, NILM techniques are relatively immature at this stage and have not reached the point where best practices can be defined. Thus, this section summarizes the most common practices and methods available in the literature and discusses the advantages and disadvantages of these methods based on the authors understanding in the field.

### 5.1. Outline of the Existing Practices for NILM Data Pre-Processing

#### 5.1.1. Balancing

A large amount of paradigms of an appliance in operation is necessary for the supervised learning algorithms to be able to detect the appliance in the total power signal with good accuracy. However, it is observed that for some of the appliances, the switch-on times (active time) are relatively small compared to the switch-off times (idle time). For example, an espresso machine is on only for a few minutes every day; thus, it is difficult to collect a large amount of representative paradigms where the appliance is on. In addition, the monitoring of load consumption in households reveals significant differences of individual habits and the daily routines of occupants. These habits and individual routines affect the usage of household appliances and, therefore, the number of events found in energy consumption data. The existing datasets in NILM are characterized as highly imbalanced. However, data balancing improves the models’ performance and alleviates overfitting at the same time. There have been observed two different kinds of imbalance in NILM datasets: (i) the imbalance that is caused by the difference in the active and idle time of appliances and (ii) the imbalance appearing because some appliance types are represented by more measurements than others (e.g., espresso machine versus air conditioner). Here, we emphasize that the majority of the commonly used datasets have a limited time duration; thus, the available training samples are few, and usually, this has an impact on the model’s performance (see Section 7).

There are various research works dealing with the first imbalance case caused by the difference in the active and idle time of appliances [53]. This can influence the performance achieved by a particular classifier trained using these data [53]. Balanced data are necessary in order to avoid the issue of bias due to a lack of an adequate number of appliance activations, which is a common problem in many NILM datasets. Every appliance should have a representative number of examples of its activation in the training learning process for supervised disaggregation algorithms. For the second imbalance case, where some appliance types are represented by more measurements than others, this could be a problem in the case of “all-in-one” models. Different techniques for handling this imbalance and avoiding biasing the classifiers during training were investigated in [53].

#### 5.1.2. Handling Sample Rates and Missing Data

High-frequency energy meters are essential in order to capture the transient events or the electrical noise generated by the electrical signals [54]. Thus, the more frequently energy consumption is measured, the more detailed is the captured information of energy consumption. However, increasing the sampling frequency will increase the data to be stored, processed, or transmitted, which in turn increases the hardware cost exponentially [22,55]. Therefore, most recent studies focus on low-sampling-frequency data, as the majority of commercial smart meters collect data usually at 0.1 Hz or up to 1 Hz to minimize the hardware cost of smart meters, their financial cost, and to address the transmission and data storage capacity limitations [55].

Most of the datasets come in a variety of sampling rates [52]; thus, in order to propose a robust NILM model that incorporates information from different datasets, it is important to have a tool to successfully re-sample the data. Using re-sampling techniques, problems related to missing data have been overcome, and with down-sampling, the overall size of a dataset can be reduced, targeting more flexible data inputs [56]. Data re-sampling filters out erroneous readings and finds the gaps in data readings, which are necessary practices in order to improve the models’ performance.

#### 5.1.3. Optimal Features’ Extraction and Selection

Feature selection is an essential step in machine learning in which a subset of relevant features or variables is identified and selected to be used in the model construction. Usually, the various values and features are selected according to their statistical importance determined by various algorithms (such as the ReliefF algorithm [57]), resulting in a meaningful feature vector. There are various studies that experimentally prove that information that is offered by more (additional) features could improve the accuracy and reliability of the NILM algorithms [39,40]. However, in most of the open-access datasets, this information is not always available. The different features that can be extracted from the acquired data are determined based on the sampling rate of power meters, that is low-frequency or high-frequency.

Low-frequency measurements: Some of the commonly used low-frequency features for load identification are the active (P)-reactive (Q) power plane (P-Q plane) [58], macroscopic transients, active power [59], and current- and voltage-based features [40,60]. Here, we highlight that although there are few works dealing with additional features for low-frequency machine learning techniques, the majority of the approaches focus solely on active power measurements, which is a variable that exists in the majority of the open-access datasets. Furthermore, there are a few studies where a set of features based on active power values is extracted. These features could include, but are not limited to, various statistical measures, such as minimum, maximum, mean, and median values, percentiles, standard deviation, skewness, kurtosis, etc.

High-frequency measurements: As regards the high-frequency steady state and transient features used in load identification [61], examples are the spectral envelope, wavelets, shape features, raw wave forms, voltage–current (V-I) trajectory, etc. High-frequency-based NILM methods found in the bibliography are based either on spectrogram analysis [54,62] or on current–voltage trajectories [63]. Rather than relying solely on time-domain analysis, in [64], a two-dimensional (2D) representation was used as the description of the power signal. Furthermore, in [62], high-frequency current data were converted to spectrograms by the short-time Fourier transform (STFT) and set as the model input. Furthermore, in [65], a V-I-trajectory-enabled transfer learning method for NILM was proposed. At first, the V-I trajectories are transformed to a visual representation in a color space, and then, a pretrained convolutional neural network is fine-tuned to perform classification on the color images of the V-I trajectories. A comprehensive review that highlights the dependence between the NILM features and the sampling rate used was provided by [4].

## 6. Machine Learning for NILM

The machine learning approaches in solving NILM problems should always keep the trade-off between the complexity of the model/architecture and the accuracy improvement. Here, we introduce a list of common dilemmas that the NILM researchers face. The solution to these dilemmas is not always obvious and depends on the data acquisition, the datasets’ availability and accessibility, the need for near-real-time NILM capabilities, the system’s scalability, as well as whether the system is able to recognize various different appliances and types of appliances [66].

### 6.1. Research Dilemmas and Conflicting Views

#### 6.1.1. Classification or Regression Model

**Figure 3:**
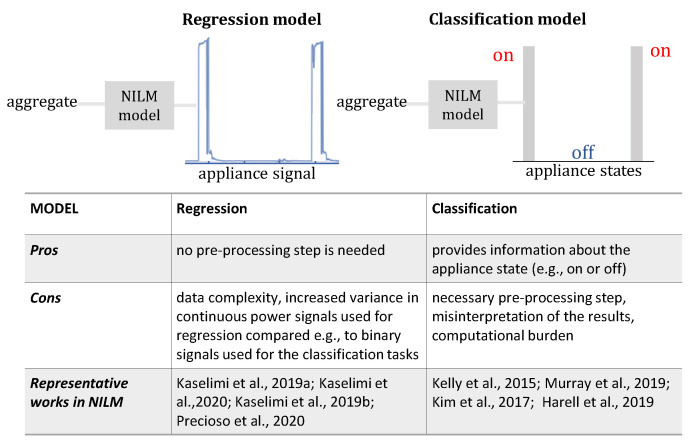
NILM as a regression or classification problem [9,18,19,26,37,39,40,68].

In the majority of NILM datasets, both the aggregated power load and the power consumption of each monitored device are included. On the contrary, the appliance switch-on events are provided only in a few datasets [67]. Thus, a regression problem to predict the consumption of each device is naturally derived from the data [68]. However, most works in NILM address the classification problem of determining whether the appliance is in operation or not, rather than estimating its consumption at each time interval. The advantages and disadvantages of the two approaches in NILM are summarized in Figure 3.

A classification problem (usually also named as event-based approaches in the literature [54]) requires a threshold or even more sophisticated event detection procedures to determine the appliance state given the continuous power load. An event detection in NILM aims to detect the times when state transition actions occur in the power consumption signal. The state transition actions normally include appliance turn-on, turn-off, speed adjustments, and function/mode changes. The event detection becomes more challenging when the appliances with the different level of energy demand are operating simultaneously, requiring high sampling data to create unique signatures and to differentiate one appliance from the others. Moreover, keeping track of the on/off timestamp, the duration of on/off, and the calculation of average load consumption during specific active periods makes the algorithms more computationally intensive [69]. In classification techniques, an accurate event detection approach is a prerequisite for precise load identification and valid power consumption estimation. Depending on how this pre-processing step is performed, the performance and interpretation of the final results may vary significantly.

Defining NILM as a regression problem obviates the intermediate step of event detection (non-event-based approaches), and the per-appliance disaggregation value is obtained directly from the results of the regression output layer.

#### 6.1.2. Multi-Target or Single-Target Model

A disaggregation model can be trained either as a single-target [37,70] or multi-target [71] regression problem or as a single-label [72,73] or multi-label [74,75,76] classification problem. Single- and multi-target NILM classification methods were explored in many works in the literature. Instead, to the best of our knowledge, multi-target regression models for disaggregation are not well-studied yet. It is worth mentioning that, in the work of [49], the authors proposed a multi-target model based on the U-Net architecture, which simultaneously performs multi-task classification and regression.

**Figure 4:**
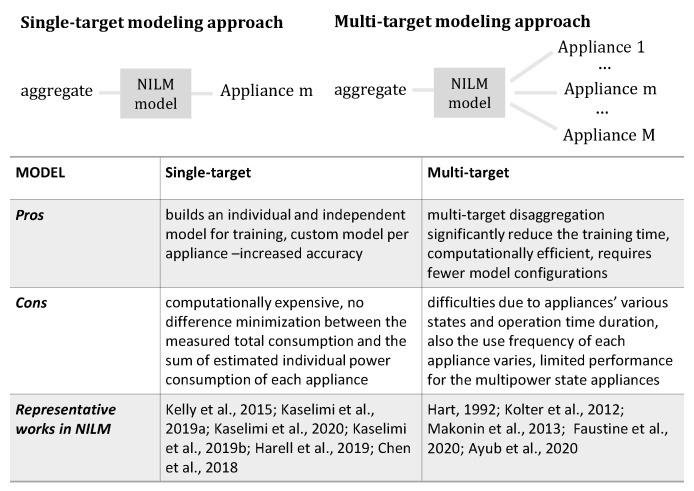
The single- or multi-target modeling approach dilemma in NILM [2,9,18,29,33,37,39,40,42,49,69].

The early works in NILM aimed at multi-target classification models (see [2,29,33]). The main difficulty arising from the multi-target approach is that the pattern and behavior of each appliance differs, so it is difficult to create a unique model able to disaggregate the main power consumption of a household simultaneously for all the individual appliances (see Figure 4). This is due to the fact that there are appliances with various states and operation time durations. Furthermore, the use frequency of each appliance varies. Given that these appliances have different frequencies of appearance, it is difficult to create the “universal” balanced dataset needed to fit each appliance’s needs. This explains why most of these models are usually formulated as sparse models [33,77,78], in order to handle the rare appliance activation events in time. Sparsity is a common problem in NILM, because the time duration that most of the appliances are in operation is relatively small compared to the time duration that the appliance is off.

Later, with the release of large amounts of datasets, supervised machine learning models that represent the nonlinear relationship between the aggregate signal and a single appliance each time (single-target model) became a method trend for solving NILM. Currently, most of the existing deep neural network models for NILM use a single-task learning approach in which a neural network is trained exclusively for each appliance. On the one hand, this is an efficient approach since the analytical models for each appliance can be developed independently of each other and transferred to unseen houses. On the other hand, in order to perform a full disaggregation into a single house, a number of different models should be trained at first and, then, be activated in order to detect how many appliances are in operation for a specific time. Furthermore, these techniques need a vast amount of data for training and houses equipped with smart plugs per appliance. The challenge here is to relax the NILM algorithms and propose models that require less amount of data leveraging, for example the advantages of semi-supervised techniques. In addition, these methods can significantly underestimate or overestimate the aggregate power consumption since they do not minimize the difference between the measured total consumption and the sum of estimated individual power consumption of each appliance. This happens because in a single-target approach, each model is trained independently of the other appliances [79]. Recently, the work of [49] suggested a multi-target U-Net network with promising performance against the traditional single-task learning, whereas in [80], a multi-target NILM algorithm was proposed using a random forest regressor.

#### 6.1.3. Supervised or Unsupervised Learning

NILM systems are categorized into supervised and non-supervised approaches depending on whether or not they require a training process prior to the model deployment on a target household. Unsupervised NILM systems do not require training, and therefore, it is expected that they will have a wider applicability. The early works in NILM mostly targeted unsupervised learning [58], as the labeled datasets were limited. These works mostly used an event detector—a clustering algorithm—and then, a transition matching stage follows, in which the on and off events belonging to the same appliance are grouped together so that the whole operation interval of each appliance can be inferred [58].

**Figure 5:**
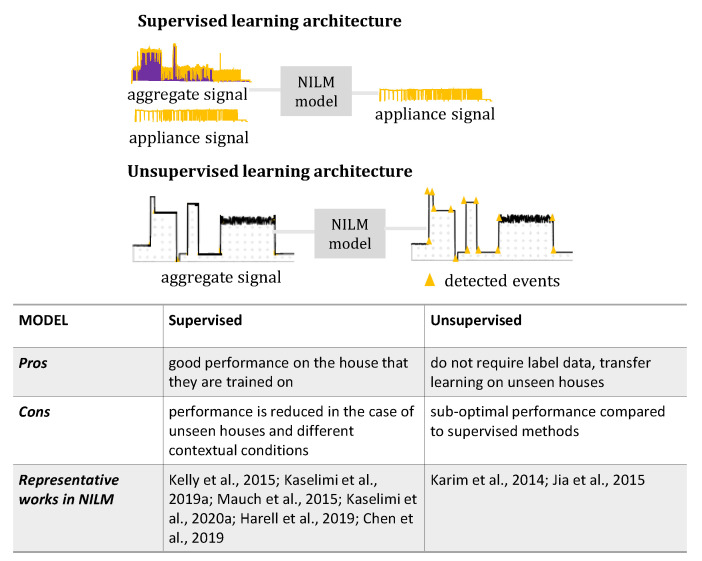
Supervised versus unsupervised NILM algorithms [9,18,32,37,40,42,58,84].

Later, with the release of large amounts of labeled datasets, supervised machine learning models became a trend. Currently, there is a variety of labeled datasets, so supervised learning is a common way of solving the NILM problem. Supervised machine learning methods work with very good performance on the house that they are trained on, but are not always transferable to unseen houses [81] and different contextual conditions. On the contrary, unsupervised NILM models usually have sub-optimal performance compared to supervised methods, but they are robust to a wide range of datasets where no training information is available. This is the reason why, even if supervised deep learning methods achieve remarkable performance, the recent works propose semi-supervised [82,83] or even unsupervised models [84], in an attempt to balance between the accuracy and robustness. While supervised NILM methods are expected to perform best on the house they were trained on, this is not necessarily the case with transfer learning on unseen houses: unsupervised NILM may be a better option. The advantages and disadvantages between these two approaches are summarized in Figure 5.

#### 6.1.4. Convolutional or Recurrent Layers

The recent increase in the availability of load data, for model training, has ignited data-driven approaches, such as deep neural networks using both convolutional neural network (CNN) and recurrent neural network (RNN) architectures [9,18,19]. The nature of the data in load disaggregation is a uni-dimensional time series that keeps track of the power consumption of each appliance in time. The NILM problem requires algorithms with the ability to process temporal information or data.

RNNs with their recurrent connections are able to refer to previous states and, therefore, are suitable models for processing sequences of input data. However, RNNs lack the ability to learn long-range temporal dependencies due to the vanishing gradient problem, as the loss function decays exponentially with time [85]. LSTM models rely on memory cells, controlled by forget, input, and output gates, to achieve long-term memorization [85]. Despite their effectiveness in capturing temporal dependencies, their sophisticated gating mechanism may lead to an undesirable increase in model complexity. At the same time, computational efficiency is a crucial issue for recurrence-based models, and considerable research efforts have been devoted to the development of alternative architectures, such as GRU networks. These have been widely proposed in NILM [19].

**Figure 6:**
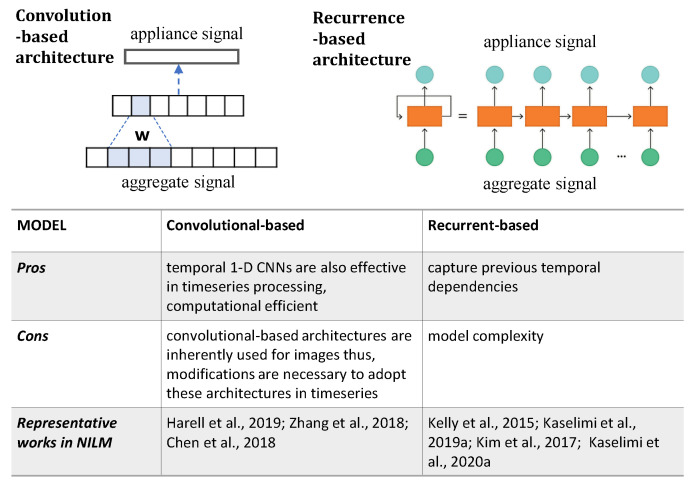
Convolutional or recurrent layers for deep learning models in NILM [9,18,26,37,40,41,42].

Causal or temporal 1D CNNs are also effective in time series processing (see Figure 6). There are various works that take advantage of the emerging advancements of the traditional CNNs and their proposed modifications, to be applied in time series problems [86]. Thus, various works have proposed causal or temporal 1D CNN to address NILM-related challenges [40]. These networks combine causal, dilated convolutions with additional modern neural network improvements, such as residual connections and weight normalization, to reduce the required computational power without performance degradation.

Alternative approaches suggest hybrid CNN-RNN architectures, which benefit from the advantages of both convolutional and recurrent layers. Representative examples of how these hybrid structures can be applied to NILM are [39,87].

#### 6.1.5. Causal or Non-Causal Models

**Figure 7:**
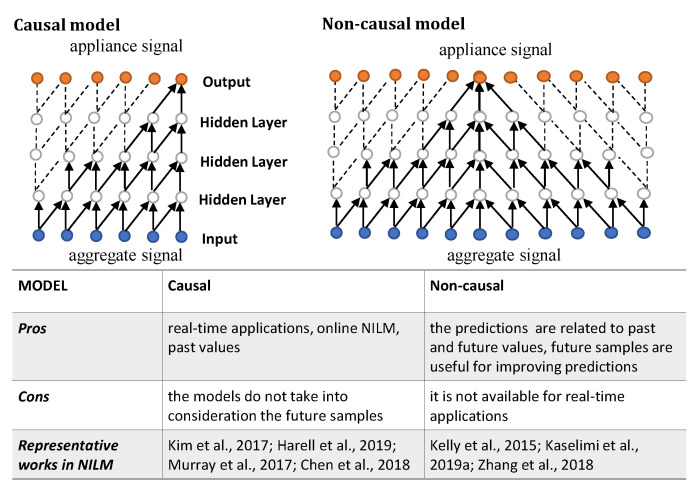
The advantages and disadvantages of the causal and non-causal NILM models [9,18,26,36,40,41,42].

As indicated in Figure 7, there are two different approaches applied for solving NILM based on causal and non-causal techniques. In the work of [40], the importance of causality in NILM was highlighted. Causal convolutional neural networks use samples from previous times steps to calculate the current output. Thus, unlike standard convolution, causal standard convolution uses the previous time step sample to predict the current result. In addition, causal dilated convolution is introduced to increase the respective field. In the causal dilated convolution, the filter is applied over an area larger than its length by skipping input values with a certain step. As stated in [40], maintaining causality is important in NILM, as it allows for disaggregated data to be made available to users in real-time, achieving on-line NILM.

In cases where causality is not necessary, non-causality is important, as the future samples are generally useful for improving predictions. For a non-causal prediction, bidirectional RNN was proposed in [18], in which a backward hidden layer was added to the standard LSTM architecture to utilize the future inputs and infer appliances’ behavior, based on both past and future samples. As regards CNN-based architectures, a non-causal (bidirectional) dilated convolution was proposed in [88]. Figure 7 shows that the bidirectional structures eliminate causality to access an equal number of samples in the past as in the future and make the prediction at the center of receptive field, which results in a larger receptive field and higher performance.

#### 6.1.6. Sequence-to-Point or Sequence-to-Sequence Techniques

**Figure 8:**
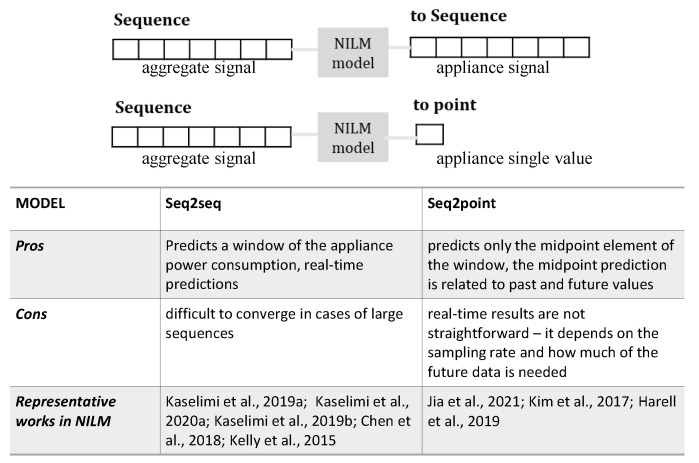
Sequence-to-sequence versus sequence-to-point approaches for NILM [9,18,26,37,39,40,42,67,88].

The sequence-to-sequence (seq2seq) and sequence-to-point (seq2point) methods achieve remarkable accuracy results for load disaggregation tasks (see Figure 8). Internally, they rely on neural networks, trained to identify the power consumption of a single appliance given a sequence of aggregate power data. In both methods, a window of (aggregate) input data is provided to a neural network, which has been trained to represent the relationship between the aggregate signal and the signal of the appliance under consideration. Thus, a sliding window is moved across the aggregate power signal and used to emit the disaggregated device-level power, either for a sequence of the same size as the input (seq2seq) or only its mid-point element (seq2point). It is noted that due to the individual consumption characteristics of most electrical devices, a separate neural network must be trained for each device. As such, it is not strictly necessary to find a sliding window size that fits all appliances equally well. However, window size is an important parameter to be estimated. There are various works claiming that the window size is directly related to the appliance type [89] and the appliance in-operation duration [9,18].

In cases where the length of input (aggregate) and output (appliance) sequence increases, applying seq2seq learning would make the training process difficult to converge. Seq2point learning has been introduced to overcome this problem [41]. Instead of training a network to predict a sequence of appliance power consumption values, seq2point only predicts the midpoint element of that sequence window. This approach could make use of all nearby regions of the input sequence, past and future, making the prediction problem easier and yielding a more accurate output. However, seq2point is somewhat extreme because every forward process of the model only yields as the output a single value, thus introducing too much computation during the inference period. Besides, the implementation of the seq2point network shows a lack of accuracy [41].

The work of [45] proposes a new perspective in the seq2seq or seq2point dilemma: a trade-off between these two approaches, i.e., the amount of computation and the difficulty level of training the neural network, by introducing a novel sequence-to-subsequence (seq2subseq) learning method.

#### 6.1.7. Uni- or N-Dimensional Problem

Usually, the data utilized in energy disaggregation are a uni-dimensional time series that monitors the total power consumption at each time, along with the respective information of the load consumption of each appliance (in the case of a supervised learning approach) [69]. Considering NILM as a time series problem, load disaggregation techniques based on sequence-to-sequence mapping are performed. Thus, given the one-dimensional input (aggregate) signal, the model learns to reconstruct the time series of a particular household appliance. Prominent examples are autoencoders as in [9] or sequence-to-sequence algorithms with recurrent layers and their variants (LSTM, biLSTM, GRU layers) [37] and, more recently, temporal 1D-CNN networks [40].

Shifting the NILM problem from the uni-dimensional discrete space to the 2D space is an alternative approach that has received some attention [90]. The authors of [91] represent the plots of the current–voltage trajectory as binary images that are fed to a CNN-based classifier in order to identify the appliances. In the paper of [63], the high-frequency aggregated current and voltage signals were transformed into two-dimensional unit cells as calculated by double-Fourier integral analysis and used as the input to a convolutional neural network for regression.

In the case where the input is neither power values (1D), nor current–voltage values (2D), but a set of different variables such as reactive power, apparent power, current values, etc. [39], for different appliance and houses, at different time steps, then a solution in the 3D (or *N*D) space can be applied. Novel deep and tensor learning (tensor decomposition) techniques [92] can also be useful to decompose the total consumption into individual appliances’ consumption values. Batra et al. [93] proposed a transferable tensor factorization approach, in which the tensor has cells that contain energy readings of the *M* houses (1st dimension) for *N* appliances (2nd dimension) and for *T* time steps (3rd dimension).

### 6.2. Trends in Machine Learning Approaches for Solving NILM

**Figure 9:**
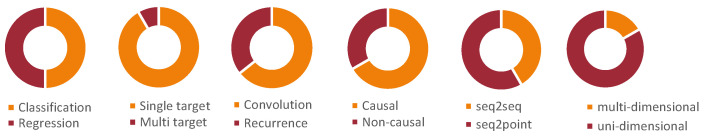
Pie charts with the final choices between the different NILM research dilemmas, based on the research works mentioned in Table 1.

Table 1 summarizes the most recent research works in NILM that dealing with the above-mentioned research dilemmas and highlights the decision/proposals of each research work with respect to the research dilemmas. As regards the supervised or unsupervised research dilemma, we already mentioned in Section 5.1.3 that, even though supervised learning algorithms are widely adopted in NILM, semi-supervised, self-supervised, and unsupervised methods have recently attracted the interest of the scientific community. In this table, we include only works related to supervised learning techniques. As indicated in Figure 9, the strongest debates are taking place between the classification or regression model and the sequence-to-point or sequence-to-sequence dilemmas. As regards the single- or multi-target model dilemma, even though until now, the most common deep learning models in NILM have been dealing with the single-target approach, recently, an increasing interest in multi-target model approaches has been observed [80].

## 7. Trustworthiness in NILM Algorithms: Can We Trust AI in NILM Problems?

One major aspect of the application of AI algorithms in NILM is how reliable their outputs are, or, in other words, how one can trust the AI outputs so that one can make reliable decisions. This opens a new research field in the machine learning society, called trustworthy AI. NILM ML-based models should be reliable in order to gain consumers’ trust, otherwise NILM-based technologies will not enable the consumers’ to change their behavior. In this section, we discuss the key papers toward trusted NILM solutions. The trustworthiness solution of an AI algorithm refers to six main aspects [96]; reliability, scalability, robustness, explainability, fairness, and privacy of the NILM algorithms.

### 7.1. Reliability

In terms of NILM, reliability implies that the model could accurately distinguish the similar appliances and avoid the probability of the misclassification and misinterpretation of the results. NILM models’ reliability is even more crucial in cases of faulty appliance detection or for real-time applications. In order to achieve this, there are a few works in the literature that propose optimization techniques for NILM deep learning models that fine-tune the algorithms to accurately detect the appliances in operation with the minimum error [18,97]. The adoption of online learning techniques is necessary for the NILM algorithms to dynamically adapt to new patterns in the appliances’ data and in contextual changes (e.g., related to environment or seasonal changes). Changes may be referring to: (i) appliance’s faulty operation, (ii) different types of appliance models (model testing in unseen houses), (iii) changing of appliance operation due to changing environmental conditions and seasonality, and (iv) entrance of a new domestic appliance into the total load. The work of [37] proposes a context-aware model that is adapted in the various conditions, resulting in an improved performance compared to traditional deep learning NILM models that are trained only once.

Under a NILM framework, it is important to deploy flexible NILM algorithms, adaptable to new appliances or appliance replacements, as in [98,99]. Here, it is worth mentioning the work of [99], which proposes a semi-supervised approach for online learning for NILM using conditional hidden Markov models (HMMs). In order to accurately detect all these changes, continual/active learning methods are essential, given that stationary pre-trained models cannot effectively deal with non-stationary appliances’ power data distributions. As regards the transferability of NILM algorithms, i.e., the ability of the algorithm to disaggregate appliance loads that have previously not been seen (or trained) by the NILM solution [19], this has been widely studied in various research works, such as in [19,20,100].

### 7.2. Scalability

Scalable AI for NILM is defined as the ability of the (i) data, (ii) NILM algorithms, and (iii) infrastructure to operate at the size, speed, and complexity required to solve the NILM problem. The challenge of making NILM models scalable is crucial mostly because the existing deep learning solutions result in models with millions of parameters and a high computational cost. On the contrary, utilities should perform a large-scale deployment to support thousands of consumers to benefit as much as possible from energy disaggregation services. Here, it is important to highlight that multi-target models (see Section 6.1.2) usually suffer from the scalability problem as the number of devices to observe rises and the inference step is computationally heavy. The current state-of-the-art NILM algorithms propose efficient techniques and models that do not require vast amounts of trainable parameters [97]. Secondly, the proposed system should be delay-free: once the appliance has been turned on, the system is able to calculate its power in near-real-time. A scalable real-time event-based energy disaggregation methodology using convolutional neural network was proposed by Athanasiadis et al. [101], whereas Krystalakos et al. [102] proposed real-time energy-disaggregation-method-based recurrent network architectures.

### 7.3. Robustness

The power signal exhibits severe nonlinearity, since the temporal periodicity of the individual appliance activation depends on contextual characteristics [103], i.e., geographic and socioeconomic parameters or even residents’ habits. This leads to diverse energy consumption patterns in households. Therefore, it is challenging to implement models with a good generalization ability that achieve high performance when tested on unseen houses. The importance of the number of houses can be explained in two ways. First, machine learning approaches for NILM can have an overfitting problem when the number of houses is not large enough. Data acquired from many houses can be crucial for a better generalization of NILM algorithms. As the number of houses increases, the number of combinations of appliances covered by the algorithm also increases, which makes NILM algorithms applicable to new houses. Secondly, the diversity of models for the same appliance type cannot be addressed by the limited open-access datasets available [104]. Kaselimi et al. [105] proposed a GAN-based framework for NILM that is robust even in the presence of noisy data input, achieving better results compared to other traditional deep learning models. Welikala et al. [106] proposed a NILM method that is robust even in the presence of unlearned or unknown appliances. In [107], a data augmentation technique was proposed in order to improve the generalization ability on new unseen data. The technique combines the on and off duration of the target appliance from various datasets, to form synthetic aggregate and sub-meter profiles.

### 7.4. Precision

In machine-learning-based approaches, the results do not converge to stable values [108]. Cross-validation is a statistical method for the evaluation of the learning algorithms and a technique to assess the generalizability of a model to unseen data [109]. During the cross-validation process, the training and validation sets cross over in successive rounds. The k-fold cross-validation is a basic form of cross-validation. These techniques provide an insight into the model’s precision level and are a necessary part of the algorithmic process to ensure the models’ stability and to define the confidence intervals of the proposed method.

### 7.5. Explainability

Understanding the outputs of the networks contributes to improving the NILM model structure, highlights the relevant features and aspects of the data used for making the decision, provides a clearer picture of the accuracy of the models (since a single accuracy number is often insufficient), as well as inherently provides a level of trust in the value of the provided consumption feedback to the NILM end-user. Murray et al. [51,110] investigated how eXplainable AI (XAI-based) approaches can be used to explain the inner workings for NILM deep learning models and examined why the network performs or does not perform well in certain cases. Explainable AI is utilized to analyze input data and address biases, especially when the NILM algorithms are tested in unseen houses, in order to improve the performance of the models [110].

### 7.6. Fairness

In the literature, fairness in AI is defined as the absence of prejudice or preference for an individual or a group based on their characteristic attributes [111]. Bias exists in many shapes and forms and is introduced at any stage of the model development pipeline. As regards data interpretation, the datasets usually suffer from historical bias, representation bias, measurement bias, temporal bias, or even, omitted variable bias. As regards the machine learning model’s deployment, algorithmic and evaluation biases are usually met in NILM techniques. Finally, human-in-the-loop approaches that consider humans as the reviewers of the model’s predictions can also introduce their own biases, when they decide whether to accept or disregard a model’s prediction. Examples of these kind of biases are social or behavioral biases.

### 7.7. Safety and Privacy

Deep-learning-based NILM models largely rely on sufficient and diverse training data gathered in centralized platforms. Even though there are plenty of meter data in different households, it is almost impossible to transmit or integrate these local user data into a centralized storage, due to limits arising from the legislation on consumer privacy and data security. In addition, even though smart meter devices collect data at a high resolution, the storage is usually performed every 15 min for the integral of consumption for privacy-preservation reasons. As a result, disaggregating time series with a time step of 15 min are much more demanding in these cases. Data security and user privacy issues have become an issue of major importance. Thus, the privacy and security of consumers’ sensitive data should be enhanced at all levels of the data processing workflow. Safety and privacy issues may imply fewer data available; therefore, additional challenges for training NILM models with good performance appear. However, recently, federated schemes have emerged as the state-of-the-art techniques in order to achieve personalized energy disaggregation with state-of-the-art accuracy, while ensuring privacy preservation for the consumer [8]. These schemes allow for federated model training, without requiring data transfer and safeguarding, because the data do not have to leave the local source premises.

## 8. Datasets, Performance Evaluation/Validation Strategy, and Open NILM Tools

### 8.1. Datasets

The existing surveys on NILM highlight the importance of selecting the right dataset. Huber et al. [112] summarized the main characteristics of the open-access datasets. In addition, in [52], a comparison between the different open-access datasets was performed. Table 2 describes the most common datasets used for NILM along with their characteristics. Here, we highlight that the selection of a dataset is directly related to the NILM method one would follow, since a dataset sets its own constraints, preventing the application of some machine learning models. For example, as indicated in Table 2, the AMPds [35] dataset has a sampling rate of 1 min. This means that this dataset cannot be used in high-frequency applications, but only for low-frequency ones. On the contrary, the BLOND [113] and EMBED [114] datasets are relevant for high-frequency models. Another attribute is the overall data time duration in each dataset. The largest ones have a duration of a few years (i.e., REFIT [36], DEDDIAG [115], and IDEAL [116]), whereas the smaller ones, only of a few days, as is the case for the REDD dataset [117]. Furthermore, most of the datasets listed in Table 3 present a limited number of houses. On the contrary, most of the current techniques for NILM require for their training a significant amount of labeled appliance power data. However, such data collection is a major bottleneck in developing robust and generalized NILM solutions. Exceptions are the HES [118], PLAID [119], and IDEAL [116] datasets. Recently, with the progress of machine learning, generative adversarial networks (GANs) are employed to synthesize appliance power signatures [120,121]. SynD [122] is an example of a synthetic dataset.

### 8.2. NILM Metrics and Evaluation

Until now, there has been no common understanding or accepted format on how to report the accuracy results in NILM [52]. However, there are a few research works that present an overview of performance evaluation metrics in NILM and the ones worth mentioning are [52,133,134,135]. Under a NILM framework, the metrics need to be reported in overall disaggregation scores (household-level) and appliance specific scores (appliance-level). The household-level metrics show the overall model accuracy and capability of disaggregating the total power signal into its component signals. This type of evaluation could be beneficial in application scenarios where a big set of houses is incorporated into a single NILM model. In this scenario, one should test the model’s accuracy at a house level and the investigate model’s transferability to different houses, through the evaluation of the model’s performance in each house separately. Furthermore, it is important to report the performance per appliance (appliance-level metrics) in order to identify the strengths and weaknesses of the different NILM algorithms. With this more detailed accuracy information, one could imagine a system that would select different algorithms depending on the context (including specific history) of the disaggregation task. As a consequence of the variety of existing load disaggregation techniques, performance evaluation has to objectively assess classification, as well as regression performance in order to enable comparability. Thus, metrics utilized in NILM can be divided into: (i) classification metrics used to evaluate the model’s performance on an appliance event detection (e.g., on–off events) and (ii) power estimation metrics applied to regression-based approaches assessing the model’s performance on appliance power signal decomposition from the total power signal [52].

#### 8.2.1. Classification—Event Detection—Metrics

The performance evaluation in this approach aims to assess the method’s effectiveness in the accurate estimation of appliance status (on/off events). Here, the most common metrics used are: the accuracy, the precision, the recall, and the F1-score [134,135]. In particular, Pereira et al. [135] analyzed experimentally the behavior of eighteen different performance metrics applied to classification NILM algorithms.

#### 8.2.2. Regression—Power Estimation—Metrics

The performance evaluation of this category (regression-based approaches) aims to assess the effectiveness of a method by comparing the observed appliance signal (ground truth) and provided estimates. Commonly used metrics are: the mean absolute error and the root-mean-squared error. Pereira et al. in their work provided a detailed list of metrics used along with their description [135].

**Figure 10:**
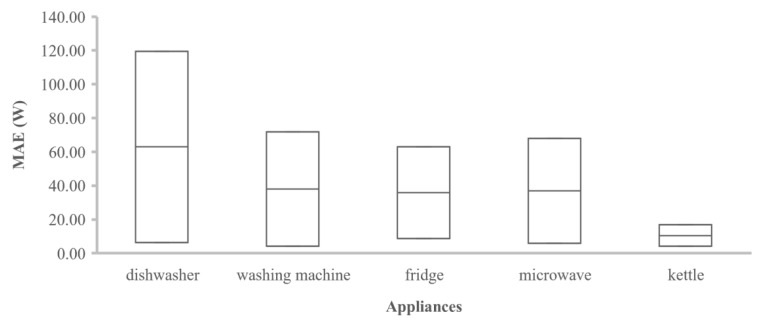
Minimum and maximum MAE error performance achieved for the top-5 commonly used appliances.

**Figure 11:**
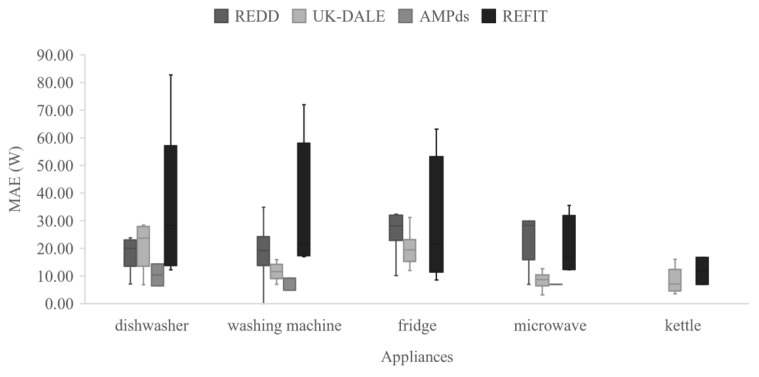
Minimum and maximum MAE error performance achieved for the top-5 commonly used appliances in the REDD, UK-DALE, AMPds, and REFIT datasets.

Table 3 presents a comparison between the common metrics used for the evaluation of the NILM algorithms in various research works. The MAE was selected for comparison purposes because it is the most common metric in the bibliography up to now. The second column indicates the datasets where the comparisons are performed. The table displays the performance errors (MAE in Watt units) for the five most-common appliances in disaggregation tasks. In bold, we highlight the minimum MAE error in which the proposed model achieves the best performance. Grey cells indicate that the disaggregators for a specific appliance are not available. The last column marks in ticks the literature works in which the models were tested for unseen houses of the same dataset. The work of [18] proposes a Bayesian-optimized bidirectional LSTM network that achieves the minimum MAE error for the dishwasher appliance in the AMPds dataset. As regards the washing machine appliance, the minimum MAE error was achieved in [39]. Murray et al. [19] proposed a convolutional-based network that achieves the highest performance in the fridge appliance for the REFIT dataset. As regards the microwave and kettle appliances. Pan et al. proposed a model based on conditional GANs with the minimum error for these appliances in the UK-DALE dataset, i.e., 3.1 and 3.6 W, respectively. Therefore, it is clear that disaggregation performance highly depends on appliance type. Figure 10 presents a summary of the minimum and maximum MAE performance errors achieved for the five commonly used appliances. The kettle seems to have the minimum MAE error compared to the other appliances, whereas the dishwasher has a larger deviation. In Figure 11, for the dishwasher appliance, the AMPds dataset achieves the best results. The same is applied for the washing machine and microwave appliances. In general, AMPds is a robust dataset; however, it lacks variability in appliances. As regards the REFIT dataset, we know a priori from the literature that this is a noisy dataset, and this is confirmed by the diagram in Figure 11, as the appliances in this dataset show the worst performance.

### 8.3. Open NILM Tools towards Commercialization

NILMTK [136] and its recently released version NILMTK-Contrib [137] comprise a common tool and the most well-known framework for benchmarking in NILM towards reproducible NILM algorithms. Its presence as an open-access toolkit and the successful implementation of various energy disaggregation algorithms have unfolded a means for comparisons of the different algorithms in the NILM research community. Furthermore, it enables researchers to observe and evaluate NILM approaches in multiple datasets that are accessible online. Except for comparing against benchmarking disaggregation algorithms, NILMTK provides dataset statistics, pre-processing tools, and NILM metrics that help towards the comparability of the various proposed methods.

Except for NILMTK, which is a combined effort towards the reproducibility and standardization of NILM algorithms, there are several works and NILM researchers that publish the code associated with their works on code repositories that are freely available. Research works worth mentioning that have been published with source code or extensive supplemental material are the following: the WaveNILM model [40], the Neural NILM model [9], and the seq2point NILM model [41].

## 9. NILM Applications

NILM techniques combined with home energy management systems (HEMSs) and ambient assisted living (AAL) technologies assist with decisions about efficient energy management [4]. For the successful cooperation of these technologies [5,6], scalable NILM methods are necessary, since they address issues arising from different user behaviors with respect to appliance usage [7]. However, there are concerns about the privacy and security of the consumers’ sensitive data (see Section 2.2). In the literature, there is an early attempt to deliver privacy-preserving and personalized NILM using federated learning technologies [8]. However, additional research work in the field should be conducted. Additionally, NILM is also used for demand-side response scenarios. Demand response provides the possibility of shifting demand away from the peak and, thus, decreasing the corresponding cost of energy [9,10]. In this context, energy disaggregation permits power supplier companies to identify a device with a high consumption rate at a peak hour in a household and send a message to the corresponding users, asking them to postpone their usage to smooth out the current peak in the demand. In this context, one can consider the works of [11,12].

Another usage of the disaggregated electrical consumption is to identify malfunctioning appliances. As an instance, the NILM approach is applied to detect the frosting cycle of a fridge with a damaged seal, which is more frequent than the normal one [3]. For instance, the possibility of utilizing NILM for anomalous behavior detection was addressed in [13], whereas the adoption of denoising autoencoders was considered in [14]. The application of NILM in home appliance malfunction detection is essential to detect problems in appliances’ operation, which can be very useful for improving the concept of self-monitoring appliances since the latter can identify potential problems in their operation and send related messages or take actions [9].

Finally, NILM solutions can be properly combined with eXplainable AI (XAI) techniques to be beneficial for the user to realize demand–response scenarios. XAI provides interpretable AI models that are capable of explaining their outcomes and provide trust in their performance [15]. Thus, we need to point out trustworthy aspects of NILM solutions and increase the acceptance of these tools by society, revealing potential perspectives and limitations. Here, explainable AI techniques combined with visual analytics could be beneficial for the user to realize demand–response scenarios.

## 10. Discussion and Conclusions

Residential NILM is an important process for various reasons. Energy disaggregation is an essential element of energy conservation, efficiency, and careful energy utilization, since it elaborates on the energy usage tendencies. The trends observed in energy usage patterns from a household can be used for security purposes, given that an anomaly might imply appliance failure or illegal use of supplied electricity. The appliance usage patterns can also be used to calculate and control the amount of carbon emissions.

Having identified and highlighted the most important research works in NILM and critically analyzed the information gathered by identifying the gaps in NILM, as the final step, we summarize the most important aspects in the NILM pipeline in order to implement effective NILM algorithms. In particular, we discuss issues related to (i) the pre-processing and the feature extraction and selection phase, (ii) the NILM model implementation phase, and (iii) the model evaluation phase.

### 10.1. Discussion on Feature Selection and Data Pre-Processing in NILM

Aggregated (total) power consumption data stemming from smart metering measurements are essential in order to implement a successful NILM algorithm. Assuming a supervised NILM scheme, except for the aggregated power measurements, additional information of appliances’ power consumption signals is also collected. The optimal feature extraction framework of Section 5.1.3 can also be utilized to improve the model performance. We highlight that the most common additional input variables reported in the literature are: (i) the time (e.g., temporal resolution), (ii) the spatial information (e.g., household’s location), and (iii) the events (e.g., appliances availability, local regulations, existence of photovoltaic (PV) panels). These additional variables are ingested into the model to increase its performance. However, most works use only a limited subset of these variables [61]. Selecting the most effective input variables for a machine learning NILM model is a challenging problem (see Section 7) and is closely related to model performance and computational cost, as discussed in [61]. To address these issues, some works propose a feature elimination process to identify the most effective feature set [61]. However, selecting a set of optimal features is constrained by the data availability and is closely related to the methods that will be used for the appliances’ identification [57].

Regarding the data sampling period, a coarse division of 1 s usually enables feature separation between macroscopic (low-frequency) and microscopic (high-frequency) components. Even though low-frequency datasets greatly reduce the ability to distinguish among different types of appliances, compared to high-frequency datasets, the first are gaining more and more ground in NILM deep learning algorithms [112].

Another important step for a successful NILM algorithm is to handle the missing data due to various causes such as metering and transmission equipment failures. In addition, large and sparse outliers, occurring due to transients, surges, and nonlinearities in the load, are also observed [138]. To handle these issues, we can apply: (i) cluster-based handling (CBH), (ii) interpolation, or (iii) omitting specific entries. All these approaches have some disadvantages that need to be considered. At first, CBH could be time-consuming and is sensitive to the parameters. A centroid-based clustering (k-nearest-neighbor approach (kNN)) could solve these issues, but it cannot manage the outlier case. Yet, density-based clustering (e.g., the OPTICS) will tackle the outlier case, but it may fail on large datasets due to numerical instabilities. Secondly, interpolation approaches (linear or not) common to fill short gaps in power time series data. If those assumption do not hold, then we end with misinformation cases. Finally, omitting entire entries can be used while preparing a training and validation set, but this approach cannot solve the on-site monitoring request.

### 10.2. Discussion on NILM Model Implementation

NILM methods are insufficiently mature, and their performance varies based on the datasets used or the appliance that is selected for disaggregation. Table 3 shows that the model proposed by [45] performs best for microwave and kettle appliances. However, in the case of dishwasher and washing machine appliances, there are other approaches that achieve better performance. Usually, while an implemented algorithm may be ideal for one appliance, its performance may not be good enough for another. Thus, we cannot claim that there is a holistic approach that is the “best” for any appliance type.

Regardless of the machine-learning-based strategy that each researcher follows, based on the research dilemmas that are summarized in Section 6, NILM researchers should have in mind that the most important aspects in NILM arise from the practical issues encountered in this demanding domain. The machine learning and deep learning community proposes an ever-increasing number of algorithms with advanced capabilities, but with increasing complexity. It is a considerable challenge to propose efficient and practical state-of-the-art NILM algorithms that, in addition, comply with the requirements of trustworthiness, as discussed in Section 7, forming feasible solutions towards commercialization. The newly introduced transformer models with attention layers for NILM require many computational resources, and scenarios with continuous learning mechanisms increase the computational complexity issues. Recently, models based on Transformer architectures have been proposed, introducing techniques for computationally efficient Transformers [139].

### 10.3. Discussion on NILM Model Evaluation

Despite a big variety of performance measures that are observed in the literature (see Section 8), it is crucial to select the metrics carefully in order to avoid the misinterpretation of the results [52]. It is important also to keep in mind when reporting the accuracy metric that the results need to be normalized. The normalization of the results allows the readers to understand the relative standings from one appliance to another and from each appliance to the overall accuracy. In addition, reporting specific scores for appliance states is not necessary because different models of appliances follow a different event detection method and divide the states in a different way. Thus, based on the method that a researcher applies, different numbers of states at different power levels are extracted. Consequently, this metric is not comparable. Thus, it is important for NILM researchers to present both classification and regression metrics in their studies, regardless of whether NILM has been previously formulated as a regression or classification problem.

## Figures and Tables

**Table 1:** List of the representative works that target all research dilemmas and the choices the researchers finally made. Only supervised learning techniques address all dilemmas and, thus, are listed in this table.

ID	Author	Title	Classification (C) or Regression (R) Model	Multi- (M) or Single- (S) Target Model	Convolutional- (C) or Recurrent- (R) Based Architecture	Causal (C) or Non-Causal (N) Model	seq2point or seq2seq	Uni- (u) or Multi- (m) Dimensional
1	J. Kelly et al., 2015 [9]	Neural NILM: Deep neural networks applied to energy disaggregation	Classification	Single	Conv./Recur.	Non-causal	seq2seq	Uni
2	J. Kim et al. 2017 [26]	Nonintrusive load monitoring based on advanced deep learning and novel signature	Classification	Single	Recurrence	Causal	seq2point	Uni
3	C. Zhang e. al., 2018 [41]	Sequence-to-point learning with neural networks for nonintrusive load monitoring	Regression	Single	Convolution	Causal	seq2point	Uni
4	K. Chen et al., 2018 [42]	Convolutional sequence-to-sequence nonintrusive load monitoring	Regression	Single	Convolution	Causal	seq2seq	Uni
5	M. Kaselimi et al., 2019 [18]	Bayesian-optimized bidirectional LSTM regression model for nonintrusive load monitoring	Regression	Single	Recurrence	Non-causal	seq2seq	Uni
6	D. Murray et al., 2019 [19]	Transferability of neural network approaches for low-rate energy disaggregation	Classification	Single	Conv./Recur.	Causal	seq2point	Uni
7	M. Kaselimi et al., 2019 [39]	Multi-channel recurrent convolutional neural networks for energy disaggregation	Regression	Single	Convolution	Non-causal	seq2seq	Multi
8	A. Harell et al., 2019 [40]	WaveNILM: a causal neural network for power disaggregation from the complex power signal	Classification	Single	Convolution	Causal	seq2point	Multi
9	M. Kaselimi et al., 2020 [37]	Context-aware energy disaggregation using adaptive bidirectional LSTM models	Regression	Single	Recurrence	Non-causal	seq2seq	Uni
10	A. Faustine et al., 2020 [49]	UNet-NILM: a deep neural network for multi-task appliances’ state detection and power estimation in NILM	Classification/Regr.	Multi	Convolution	Causal	seq2point	Uni
11	L. d. S. Nolasco et al., 2021 [94]	DeepDFML-NILM: a new CNN-based architecture for detection, feature extraction, and multi-label classification in NILM signals	Classification	Single	Convolution	Causal	seq2point	Uni
12	W. Yang et al., 2021 [95]	Sequence-to-point learning based on temporal convolutional networks for nonintrusive load monitoring	Regression	Single	Convolution	Causal	seq2point	Uni

**Table 2:** An overview of NILM datasets. The table summarizes the year of the release for each dataset, the number of houses included, as well as the duration of the dataset and the measured variables. Furthermore, the last common refers to notes worth mentioning for each dataset.

Dataset Name	Year	Country	House No.	Duration	Variables	Aggregate Sampling Rate	Appliance Sampling Rate	Comments
REDD [117]	2011	US	6	a few months	current, voltage	1 Hz, 15 kHz	1/3 Hz	first released and most-used
BLUED [123]	2011	US	1	8 days	current, voltage	12 kHz	-	allows for analysis in both the time and the frequency domains
HES [118]	2012	UK	251	1 year	active power	2–10 min	2–10 min	number of houses
AMPds [35]	2013	CA	1	1 year	current, voltage, pf, real, reactive, and apparent power	1 min	1 min	multiple variables
BERDS [124]	2013	US	1	1 year	active, reactive, and apparent power	20 s	20 s	public building of the University
iAWE [125]	2013	IN	1	73 days	current, voltage, active, reactive, and apparent power	1 s	1 s	contains electricity, gas, and water consumption data
DRED [126]	2014	NL	1	6 months	active power	1 Hz	1 Hz	indoor and outdoor temperature, wind speed, humidity, precipitation, and occupancy information
ECO [127]	2014	CH	6	8 months	active reactive power	1 Hz	1 Hz	occupancy information of the monitored household
GREEND [128]	2014	IT/AT	9	1 year	active power	1 s	1 s	cross-country dataset
PLAID [119]	2014	US	60	summer of 2013 and winter of 2014	current, voltage	-	30 kHz	3 versions PLAID 1 (2014), PLAID 2 (2017), and PLAID 3 (2018), which include also aggregate measurements
REFIT [36]	2015	UK	20	2 years	active power	8 s	8 s	corrupted-with-noise version of the dataset
UK-DALE [67]	2015	UK	5	1 to 2.5 years	current, voltage	6 s, 16 kHz	6 s	long duration
COOLL [129]	2016	FR	1	-	current, voltage	-	100 kHz	high-frequencysampled electrical signals for appliance identification
BLOND [113]	2018	DE	1	213	current, voltage	50 kHz	6.4 kHz	building-level office environment dataset
EMBED [114]	2019	US	3	14–21 days	active, reactive power	12 kHz	12 kHz	aggregate power files, fully labeled appliance event timestamps, and plug load consumption for a variety of monitored appliances
SynD [122]	2019	AT	1	180 days	active power	5 Hz	5 Hz	synthetic energy dataset
DEDDIAG [115]	2021	DE	15	<3.5 years	active power	1 Hz	1 Hz	long duration
IDEAL [116]	2021	UK	255	<2 year	active power	1 s	1 s	electricity and gas sensor data along with a diverse range of relevant contextual data from additional sensors and surveys

**Table 3:** NILM researchfrom a practical perspective. Datasets utilized, performance achieved per appliance, and adaptability of the NILM algorithms. In cases where the unseen house option is checked, the performance in the table refers to unseen houses of the same dataset that are used only for training (RD:REDD, RF:REFIT, AM:AMPds, UK:UK-DALE).

		Top-5 Common Appliances’ MAE (W)						
A/A	Dataset	Dishwasher	Washing Mach.	Fridge	Microwave	Kettle	Overall MAE (W) per Dataset	Unseen House
[9]	UK	24.0	11.0	18.0	6.0	6.0	22.0	√
[41]	RD/UK	20.0/27.7	18.4/12.6	28.1/20.9	28.2/8.7	-/7.4	15.5 /23.6	√
[42]	RD	12.8		32.0			-	√
[18]	AM	6.4	9.2				-	
[19]	RD/RF	119.4/82.74	-/71.9	10.1/8.6	68.0/35.5		-	√
[39]	AM	14.3	4.8				-	
[130]	RD/UK	15.9/13.5	20.6/11.0	22.9/15.3	15.9/8.6		18.8 /10.9	√
[37]	RD/RF/AM	7.1/31.3/-	-/21.8/9.2		6.9 /-/-		-	√
[49]	UK	6.8	11.5	15.2	6.5	16.0	11.2	√
[20]	RD/UK/RF	20.0/27.7/12.2	18.4/12.6/16.9	28.1/20.9/20.0	28.2/8.7/12.7		23.7/15.5/13.7	√
[95]	UK	23.3		16.4	12.6	4.1	-	√
[45]	UK	13.5	7.1	11.9	3.1	3.6	7.8	√
[131]	RD/UK/RF	23.8/28.4/15.4	19.9/15.9/17.9	31.3/22.3/23.2	29.9/9.7/12.2	-/7.7/6.9	-	√
[132]	UK/RF	51.3/28.2	25.9/44.0	31.1/63.1	64.5/20.7	13.9/16.7	-	√
[48]	RD/UK	20.5/16.2	34.9/6.9	32.4/25.5	17.6/6.96	-/6.8	26.4/12.4	√

## Data Availability

References for the described datasets can be found in Section 8.
